# Supplementary material for: Improvement of Arterial Stiffness One Month after Bariatric Surgery and Potential Mechanisms
Source: J Clin Med. 2021 Feb 10;10(4):691. doi: 10.3390/jcm10040691 (PMC7916665; doi:10.3390/jcm10040691)
Supplement: Supplementary file 1 [file jcm-10-00691-s001.pdf]

SUPPLEMENTARY MATERIALS

Supplementary Table S1. Changes in anthropometric, blood pressure and arterial stiffness parameters one-month after bariatric surgery according to the type of surgery.

	**Roux-en-Y gastric Bypass (n = 27)**			**Sleeve Gastrectomy (n = 20)**			***p* Between Groups**
**Parameter**	**Baseline Mean ± SD**	**1 month Mean ± SD**	***p***	**Baseline Mean ± SD**	**1 month Mean ± SD**	***p***	
Anthropometric parameters							
**Body weight, kg**	117.4±15.8	104.2± 13.6	**<0.001**	119.1± 24.1	105.0±15.2	**<0.001**	0.448
**Waist circumference, cm**	130.9 ± 8.2	122.9 ± 7.1	**<0.001**	132.4 ± 13.7	121.7 ± 13.6	**<0.001**	0.106
Arterial stiffness							
**24h-PP, mmHg**	45.1 ± 6.5	42.7 ± 7.3	**0.047**	49.8 ± 7.7	46.2 ± 6.5	**0.006**	0.434
**24h-PWV, m/s**	6.31 ± 1.0	6.06 ± 0.92	**0.011**	6.76 ± 1.04	6.49 ± 1.02	**<0.001**	0.818
**AIx@75, %**	25.1± 6.1	21.7 ± 7.0	**0.004**	26.8 ± 8.9	24.0 ± 7.3	**0.052**	0.723
Blood pressure and other hemodynamic parameters							
**24h-SBP, mmHg**	119.0 ± 11.3	113.5 ± 11.1	**0.005**	122.8±13.6	115.5 ± 8.2	**<0.001**	0.455
**24h-DBP, mmHg**	73.9 ± 9.1	70.7 ± 8.5	**0.006**	73.1 ± 7.8	69.4 ± 3.9	**0.009**	0.759
**24h-HR, bpm**	73.1 ± 7.7	66.9 ± 7.6	**<0.001**	73.4 ± 9.2	66.6 ± 8.2	**<0.001**	0.491
**Cardiac output**	4.6 ± 0.6	4.5 ± 0.5	0.057	4.7 ± 0.6	4.5 ± 0.5	**0.001**	0.637

AIx@75 = augmentation index at 75 beats/minute; **DBP** = diastolic blood pressure; **HR** = heart rate; **PP** = pulse pressure; **PWV** = pulse-wave velocity; **SBP** = systolic blood pressure.

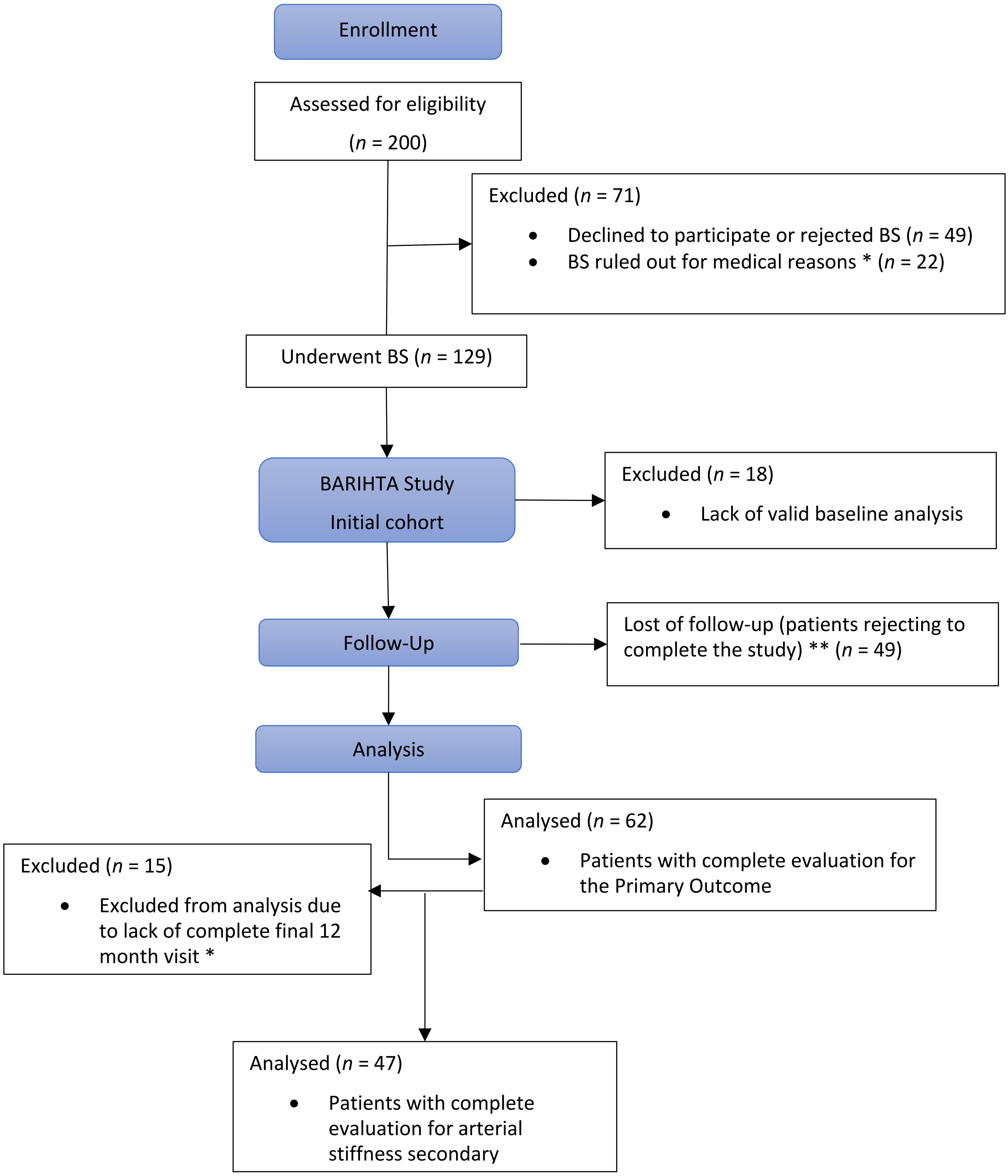

Supplementary Figure S1. Flowchart for participants in the BARIHTA Study. BS = Bariatric surgery. * None of them was excluded because of heart failure or other cardiac disease. ** Most patients rejected to complete the study because of complains about wearing the 24h-Mobile-O-Graph device or due to work reasons (having achieved work reinsertion after losing weight)

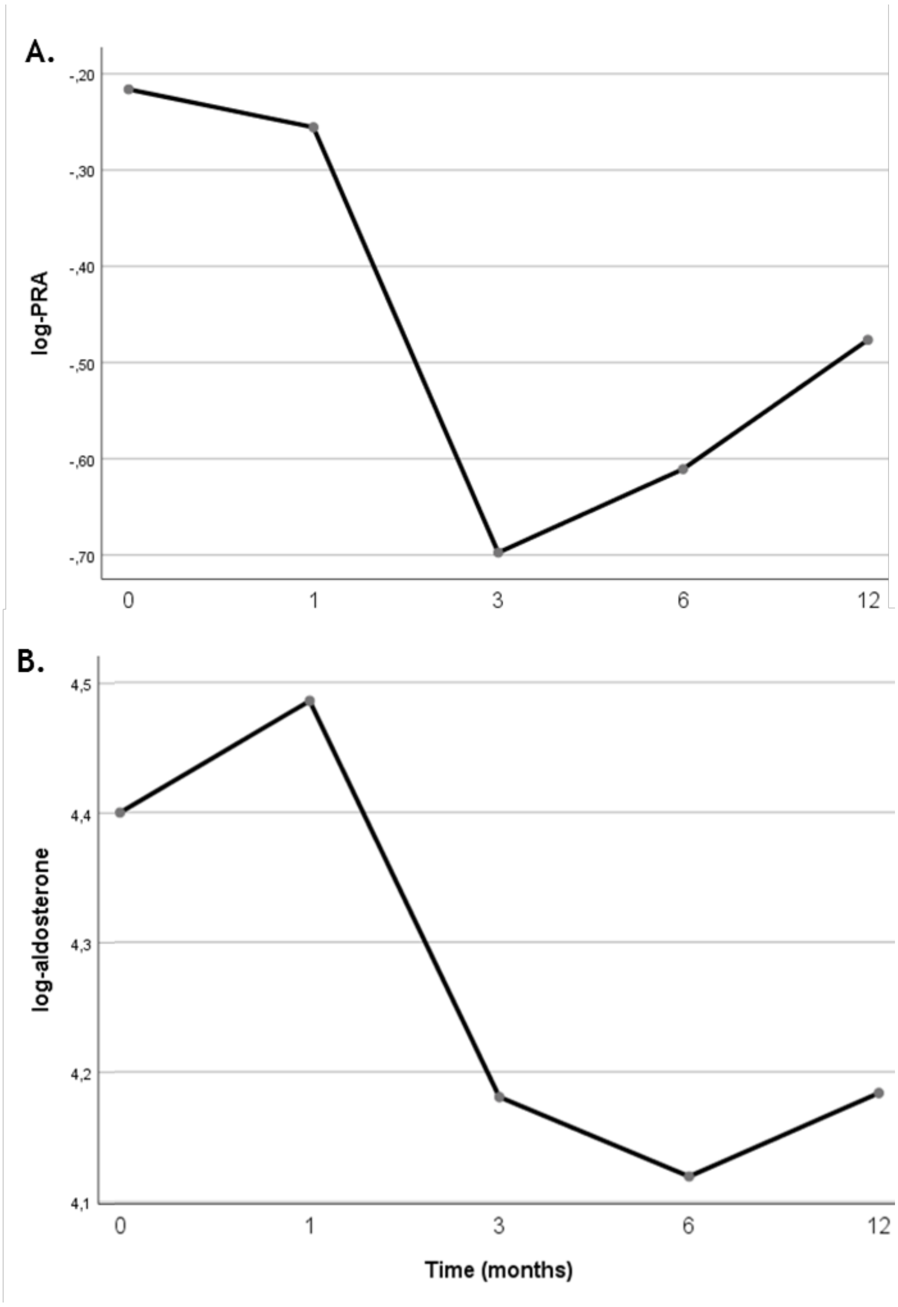

Supplementary Figure S2. (A) Variation of plasma renin activity (log-transformed) levels at follow-up. (**B)** Variation of plasma aldosterone (log-transformed) concentration at follow-up. **log** = log-transformed; **PRA** = plasma renin activity
